# F_1_·F_o_ ATP Synthase/ATPase: Contemporary View on Unidirectional Catalysis

**DOI:** 10.3390/ijms24065417

**Published:** 2023-03-12

**Authors:** Tatyana V. Zharova, Vera G. Grivennikova, Vitaliy B. Borisov

**Affiliations:** 1 Department of Biochemistry, Faculty of Biology, Lomonosov Moscow State University, 119234 Moscow, Russia; 2 Belozersky Institute of Physico-Chemical Biology, Lomonosov Moscow State University, Leninskie Gory, 119991 Moscow, Russia

**Keywords:** membrane protein, biophysics, molecular bioenergetics, inhibition, F_o_∙F_1_-ATP synthase/ATPase, reversibility of enzyme catalysis

## Abstract

F_1_·F_o_-ATP synthases/ATPases (F_1_·F_o_) are molecular machines that couple either ATP synthesis from ADP and phosphate or ATP hydrolysis to the consumption or production of a transmembrane electrochemical gradient of protons. Currently, in view of the spread of drug-resistant disease-causing strains, there is an increasing interest in F_1_·F_o_ as new targets for antimicrobial drugs, in particular, anti-tuberculosis drugs, and inhibitors of these membrane proteins are being considered in this capacity. However, the specific drug search is hampered by the complex mechanism of regulation of F_1_·F_o_ in bacteria, in particular, in mycobacteria: the enzyme efficiently synthesizes ATP, but is not capable of ATP hydrolysis. In this review, we consider the current state of the problem of “unidirectional” F_1_·F_o_ catalysis found in a wide range of bacterial F_1_·F_o_ and enzymes from other organisms, the understanding of which will be useful for developing a strategy for the search for new drugs that selectively disrupt the energy production of bacterial cells.

## 1. Introduction

H^+^-transporting two-sector ATPases (EC 7.1.2.2; systematic name: ATP phosphohydrolase (H^+^-transporting), also named F-type ATPase) are large (more than 500 kDa), multi-subunit protein complexes found in energy-transducing membranes in bacteria, chloroplasts, and mitochondria. They couple either ATP synthesis from ADP and phosphate or ATP hydrolysis to the consumption or production of a transmembrane electrochemical gradient of protons, known as the proton motive force (*pmf*).

ADP + P_i_ + *pmf* ↔ ATP + H_2_O

F_1_·F_o_-ATP synthases/ATPases (for convenience, in this review, called F_1_·F_o_) belong to the family of rotary ATPases, which, besides the F-type ATPases, also include the eukaryotic vacuolar (V-type) ATPases and the A-type ATPases of archaea and some extremophilic bacteria. V-type ATPases use the energy of ATP hydrolysis to generate ion gradients across secretory membranes, and A-type ATPases generate ATP from a proton gradient like the F-type but may also work as ATP-driven ion pumps such as the V-type, to which they are more closely related. This review focuses on recent progress in understanding the regulatory mechanisms of F-type ATPases, mainly bacterial F_1_·F_o_.

All F_1_·F_o_ from various sources have a common structure: they consist of two main parts, the hydrophilic F_1_ and the hydrophobic F_o_ complexes. For most organisms routinely used in research, structures of F_1_ have been established, mainly by X-ray analysis [1,2,3], most detailed for mitochondrial [4] and yeast [1] F_1_·F_o_ and less for bacteria [4]. The hydrophobic F_o_ structure is less susceptible to crystallization and is being studied by intensively developing electron cryomicroscopy (cryoEM) [5,6]. This method allowed obtaining a complete structure of F_o_ from various organisms, including complexes from bacteria [5]. Bacterial F_1_·F_o_ can be subjected to various genetic modifications, and by using this approach, most of the functionally important enzyme residues have been identified (mainly in *Escherichia coli* mutants). The study of the universal F_1_·F_o_ rotational mechanism on single molecules using biophysical methods made it possible to characterize the functional properties of bacterial enzyme complex [7,8]. In this review, we will mainly focus on bacterial F_1_·F_o_. Considering the growing interest in F_1_·F_o_ as targets for antimicrobials [9,10,11,12,13], in particular as anti-tuberculosis drugs [14,15], we will also discuss the strategy for developing drugs selectively affecting the bacterial cell energetics.

## 2. Common Subunit Composition and Function of F_1_·F_o_

**Figure 1:**
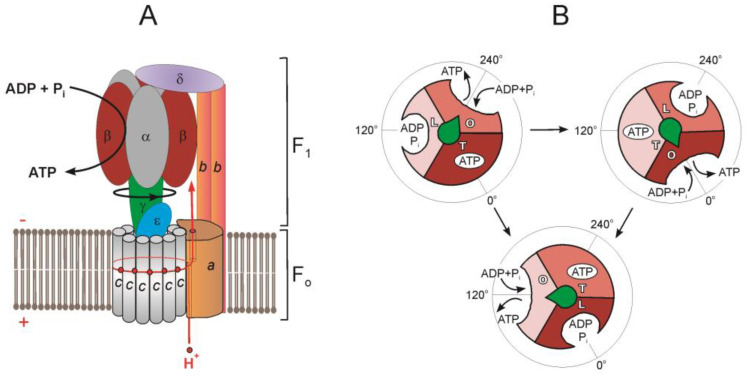
Schematic illustration showing the arrangement of subunits in F_1_·F_o_-ATP synthase/ATPase (*E. coli*) (**A**) and chemomechanical coupling scheme of F_1_·F_o_-ATPase (**B**). (**A**) Shown are subunits α (grey), β (brown), γ (green), ε (blue), *c* (grey), *a* (light brown), *b* (orange), and δ (purple). F_1_ and F_o_, ATP synthesis reaction and direction of γ-subunit rotation during ATP synthesis are marked. The proton pathway is depicted in red. The position of the half channels in *a* subunit is shown with a thin line. (**B**) Scheme of the F_1_-ATPase rotary catalytic mechanism. Each αβ subunit pair is shown in brown, light brown, and beige. Binding site occupation marked with letters: O, *open*; L, *loose*; and T, *tight*. The γ-subunit is depicted as a green arrow, and, for clarity, the other subunits are not shown. For a more detailed scheme with the indication of substages proposed for different bacterial and eukaryotic F_1_·F_o_, see review by Noji and Ueno [8].

The basic structure of the currently known F_1_·F_o_ is composed of eight canonical types of subunits: F_1_ is composed of five types of subunits α, β, γ, δ, and ε, whereas F_o_ is composed of three types of essential subunits, *a*, *b,* and *c* [16] (Figure 1A). The F_1_·F_o_ of most bacteria contains only these subunits, with the exception to date being α-proteobacteria, which have an additional F_1_ subunit called ζ [17,18]. The structure of chloroplast or mitochondrial F_1_·F_o_ may also contain other subunits [6,19].

The α and β subunits are assembled in the form of hexameric ring 3α3β that alternates α and β and surrounds the central stalk, which in prokaryotes consists of γ and ε subunits [1,4,16].

F_o_ is a complex of polypeptides that includes an *a* subunit and a rotary *c*-ring immersed in a coupling membrane [4,20]. The *a* subunit, the largest of the hydrophobic F_o_ subunits, consists of transmembrane α-helices oriented perpendicular to the plane of the membrane [20]. The *c*-ring stoichiometry is species-specific and ranges from 8 to 17 subunits [4].

F_o_ is connected to F_1_ by the central and peripheral stalks. The central stalk comprises the γε-subcomplex firmly attached to the *c*-ring forming the enzyme rotor [16,21]. The peripheral stalk in bacteria consists of *a*, 2*b,* and δ subunits (Figure 1A). The *a* subunit is membrane-bound and acts as a collar around the *c*-ring. One of the functions of the *a* subunit is structure, as it anchors the 2*b* dimer into the membrane phase. Two identical (for example, in *Bacillus* sp. PS3 and *E. coli* [16,22]) or different (for example, in *Paracoccus denitrificans* [23] and *Mycobacterium smegmatis* [9]) *b* subunits, having the form of long single α-helices, diverge in the membrane, pressing the *a* subunit to the *c*-ring. Leaving the membrane, the 2*b* dimer reaches over the 3α3β hexamer, and its opposite end interacts with the δ subunit, linking 2*b* to F_1_ [4,22]. In *E. coli*, the N-terminal domain (NTD) of the δ subunit interacts with the N-terminal region of the α subunit and segments of the *b* subunit [16]. The 2*b*δ peripheral stalk holds the 3α3β-hexamer as a part of the stator. In mycobacteria and many other eubacteria, the peripheral stalk has a special structure—it consists of two proteins, *b’* and *b*δ, where the *b*δ subunit arose as the result of the covalent fusion of the individual *b* and δ subunits [9].

It was previously assumed that the central and peripheral stalks provide a rigid connection between F_1_ and F_o_. Now, due to cryoEM data, F_1_·F_o_ is assumed to be a mobile dynamic structure, and catalysis requires significant conformational changes of multiple subunits [24,25,26]. The structurally simpler bacterial peripheral stalk of F_1_·F_o_ turned out to be more flexible than the mitochondrial one [4,25,27]. Subunit δ (OSCP in mitochondrial F_1_·F_o_) allows ATP synthase to adopt different rotational states acting as a hinge [28]. The NTD that connects to the three α subunits rotates relative to the C-terminal domain (CTD) by 20 to 30° on a flexible single-polypeptide stretch connecting the two domains [4]. Subunits in the central stalk may show flexibility during rotation under strain [16].

## 3. F_1_·F_o_ Rotary Catalysis

F_1_·F_o_ catalyzes the synthesis of ATP by consuming energy of *pmf* generated by the respiratory chain. From the side of the membrane with a higher H^+^ concentration, protons are translocated to the other side of the membrane through two half-channels located in the F_o_ at the contact interface between the *c*-ring and the *a* subunit [20]. Proton transfer through the hydrophobic environment of the lipid bilayer between half-channels is enabled by the conserved acidic residues in the *c* subunits and by the *c*-ring rotation, which transfers a proton from one half-channel to the second one before its release (Figure 1A). The conserved arginine residue generates a positive charge on the surface of the *a* subunit in the region of its contact with the *c*-ring. This positive charge prevents the short circuit, allowing protons to pass from one half-channel to another without the *c-*ring rotation [25,26,27,28,29].

The proton flow through the half-channel system is favored by the electrochemical gradient and will support the clockwise rotation of the *c*-ring (CW) toward ATP synthesis. Since the *c*-ring is physically attached to the central γε complex, the net result of its rotation is a conduction of mechanical CW rotary motion to the γ subunit of F_1_. Once the γ subunit starts rotating in the CW direction, the asymmetry of the γ subunit causes the mechanical energy generated by the rotation of the *c*-ring to be transferred to the 3α3β subunits of F_1_ [8,30]. F_1_ contains three active sites on the interfaces between β and α subunits [1]. The α and β subunits, depending on the bound nucleotide, can be in three different conformational states: open βE (no nucleotide), loose βDP (bound ADP) and tight βTP (bound ATP) [31]. In the binding-change mechanism proposed by Boyer [30], the physical position of the γ subunit determines the conformational states of α and β [31], and the γ subunit tightly attached to the F_o_ rotor causes the α/β subunits to adopt different conformations with different binding affinities for ATP or ADP and phosphate [8].

The binding-change mechanism was experimentally confirmed by direct observation of the central stalk rotation in single-molecule experiments with *Bacillus* sp. PS3 enzyme that demonstrated consumption of three ATP molecules for each 360° rotation of the bacterial F_1_·F_o_ [8]. Hydrolysis of one ATP molecule in the catalytic center of the β subunit led to a discrete rotation of the γ subunit by 120° relative to the 3α3βδ subcomplex [8] (Figure 1B). Each 120° turn is divided into two substeps: the first, an 80° rotation, is due to the binding of ATP (binding dwell), and the second, a 40°, is due to the bound ATP hydrolysis (catalytic dwell) [6,7]. The catalytic dwell conformation was observed in the earliest crystallographic structures. The binding dwell was detected during structural analysis of isolated enzyme preparations obtained by cryoEM. This method makes it possible to separate proteins according to these conformations, and also enables direct visualization of the rotational cycle at the structural level [6,8,16,19,32]. Substeps of catalytic cycles may vary from one organism to another [8,33]. For example, human mitochondrial F_1_ also demonstrates three 120° turns of the γ subunit, but each 120° turn includes three substeps: 65°, 25°, and 30°, due to ATP binding, phosphate release, and hydrolysis of bound ATP [34]. It is suggested that the number of rotational substeps correlates with the number of *c* subunits in the ring: the smaller the *c*-ring, the more dwells observed during a complete ring rotation [33].

On the other hand, the chemomechanical scheme of the *P. denitrificans* F_1_·F_o_ (*Pd*F_1_·F_o_) was found to differ from that of other known bacterial or eukaryotic enzyme complexes. Single-molecule experiments showed that during ATP hydrolysis, rotation of the *P. denitrificans* F_1_ (*Pd*F_1_) exhibited three 120° dwells per rotational cycle, without any obvious substeps, in contrast to all other known F_1_·F_o_. An analysis of the dwell time between steps showed that *Pd*F_1_ performs binding, hydrolysis, and possible release of the product in the same rotational position [33].

## 4. Reversibility of F_1_ F_o_ ATP Synthase Reaction and the Problem of Preventing Wasteful ATP Hydrolysis

Most researchers in this field believe F_1_·F_o_ to be fully reversible: F_1_·F_o_ are able to rotate their rotor in both the clockwise direction (when viewed from F_o_ to F_1_), if they function as ATP synthases [35], and in the counterclockwise (CCW) direction if they work as ATPases [8,29]. The direction of the reaction changes when *pmf* drops, for example, during anoxia in mitochondria or in the dark in chloroplasts. The bacterial F_1_·F_o_ is also assumed to be fully reversible [4]. They work in either direction depending on growth conditions. Bacteria use *pmf* across the plasma membrane, generated by the respiratory chain, to synthesize ATP from ADP and phosphate during aerobic growth. Under anaerobic conditions, bacteria generate ATP by glycolysis and fermentation. When *pmf* partially or completely dissipates due to the lack of oxygen or an alternative terminal electron acceptor, or during uncoupling, F_1_·F_o_ hydrolyzes the formed ATP to restore the membrane potential, which then can be used to activate other important cellular functions, such as chemotaxis and secondary solute transport.

If the ATP synthase reaction is reversible [35], a halt to ATP synthesis must also turn off the enzyme in order to prevent uncontrolled hydrolysis of ATP since, under physiological conditions, cells need to maintain a high ATP/ADP ratio. Indeed, in most organisms, the CCW rotation of F_1_·F_o_ is preferentially inhibited by several mechanisms to avoid energy dissipation by wasting the intracellular ATP pool.

Two types of ATP hydrolysis inhibition are known: the so-called ADP(Mg^2+^)-inhibition and inhibition by natural inhibitor proteins. ADP(Mg^2+^)-induced inhibition is inherent in all bacterial and eukaryotic forms of the enzyme studied so far [36,37,38]. In mammals and yeast, F_1_·F_o_ inhibitory factor-1 (IF1 protein) is responsible for the inhibition of ATP hydrolysis. It binds to the enzyme upon *pmf* collapse and inhibits it [39,40]. In phototrophic organisms, the formation of a disulfide bridge in the γ subunit prevents ATP hydrolysis [41,42]. In some bacteria, the ε subunit can change its conformation and integrate into the 3α3β hexamer, blocking ATP hydrolysis [24,43]. In α-proteobacteria, inhibition of ATP hydrolysis appears to be achieved by the ζ subunit [23,44,45].

### 4.1. ADP(Mg^2+^)-Inhibition

It has long been known that the preincubation of soluble F_1_ or membrane-bound F_1_·F_o_ with very low amounts of ADP or ATP, almost equal to the concentration of F_1_, in the presence of Mg^2+^, causes the complete disappearance of ATP hydrolase activity [46]. This phenomenon is referred to as ADP(Mg^2+^)-inhibition [37,46]. It is assumed that ADP(Mg^2+^)-inhibition is due to the specific ADP binding (the presence of Mg^2+^ is obligatory) in the F_1_ active site localized on one of the αβ pairs. Removal of ADP from the enzyme preparations by treatment with phosphoenolpyruvate + pyruvate kinase and removal of Mg^2+^ in the presence of EDTA causes complete but slow (tens of minutes) activation of F_o_∙F_1_ ATP hydrolase activity [36,47]. ADP(Mg^2+^)-inhibited enzyme can be activated by detergent lauryl dimethylamine oxide (LDAO) [48,49] and selenite [50] anions. Azide stabilizes the ADP(Mg^2+^)-inhibited form of F_1_·F_o_ and prevents enzyme activation [36,51]. It should be stressed that membrane energization leads to the rapid activation of the ADP(Mg^2+^)-inhibited hydrolytic activity of F_1_·F_o_ in almost all organisms studied [52,53,54].

This type of inhibition of ATP hydrolysis is caused by permanent occupancy of the catalytic site by ADP(Mg^2+^) without P_i_ [55,56]. Experiments with single molecules of *Bacillus* sp. PS3 F_1_ showed that ADP(Mg^2+^)-inhibition stopped rotational catalysis at the angle corresponding to the catalytic dwell, and the activation of the enzyme required a 40° rotation in the direction of ATP hydrolysis by mechanical action or by thermal fluctuation [57].

The degree of ADP(Mg^2+^)-inhibition varies in different organisms; ATP hydrolysis is significantly inhibited in *Bacillus subtilis* [58,59] and very strongly inhibited in *P. denitrificans* [49,54] compared to other bacterial F_1_·F_o_. The strength of inhibition correlates with the occupancy of the catalytic site by ADP(Mg^2+^): for an enzyme with a strongly inhibited ATPase activity from *Caldalkalibacillus thermarum* and *M. smegmatis*, complete occupancy is shown, and for an enzyme with incomplete inhibition from *Fusobacterium nucleatum,* only partial occupancy is shown [3].

### 4.2. Natural Inhibitor Proteins

The second mechanism of ATP hydrolysis inhibition is implemented with the help of natural inhibitor proteins: the ε subunit in bacteria [60] and IF1 in mitochondria [61]. This type of inhibitor also includes the ζ subunit of α-proteobacteria [62].

It was shown in the pioneering works that the removal of the ε subunit from F_1_ is accompanied by the activation of the enzyme ATPase activity. The structure of the ε subunit was solved for the *E. coli* model. It was found that its NTD is folded into a globular 10-strand β-sandwich, and the C-terminal domain (CTD) contains two α-helical regions connected by a flexible linker and lying next to the β-sandwich. In cross-linking and crystallographic studies, large conformational changes of these α-helices were observed. When two α-helices in CTD are parallel, in a “folded state” and spatially localized on the β-sheet rigid domain, the ε subunit assumes a compressed “hairpin conformation” and is in the so-called “down” conformation. Conversely, when these helices are arranged sequentially, they spatially move away from the rigid β-sheet and stretch parallel to the γ subunit, reaching the 3α3β catalytic hexamer. In this position (“extended” or “up” conformation), the CTD penetrates into the cavity of the 3α3β ring, wraps the γ subunit, blocks the rotation of the central stalk and inhibits ATP hydrolase activity. Upon transition to the “down” conformation, the CTD subunit ε is displaced from the 3α3β ring. As a result, it stops inhibiting ATP hydrolysis and promotes ATP-dependent *pmf* generation [60,63,64].

It was found that the *Bacillus* sp. PS3 ε subunit contains a nucleotide binding site, and ATP binding initiates conformational transitions from the inhibitory “up” to the “down” conformation of the “hairpin” [65,66]. Thus, ATP controls the conformational state of the ε subunit: at a relatively high concentration of intracellular ATP, the nucleotide binds to the ε subunit, and its folded compact structure is stabilized without preventing F_1_·F_o_ from rotating in the direction of hydrolysis. At a relatively low intracellular ATP concentration, the ε subunit assumes an “up” conformation and, upon contact with the γ subunit, controls the CCW rotation of F_1_·F_o_ and inhibits hydrolysis [60]. CryoEM studies of *Ec*F_1_·F_o_ showed that ε subunit CTD is either only in an elevated state or, after exposure to an excess of ATP(Mg^2+^), in a lower state or in an “intermediate” state [24].

Although ATP-dependent autoinhibition of F_1_·F_o_ by the CTD of the ε subunit has been shown for some bacterial species [66,67], this regulatory mechanism likely is not conserved in other genera of bacteria [3,68]. Thus, the ε subunit of *C. thermarum* is in the “down” conformation with the ATP and Mg^2+^ bound [67]. The mycobacterial ε subunit is shortened and unable to bind ATP, but it is also in the “down” conformation [69,70]. However, F_1_·F_o_ of these bacteria hydrolyze ATP at low rates. In the sequence of the ε subunit of F_1_·F_o_ in such α-proteobacteria as *P. denitrificans*, *Rhodobacter capsulatus*, and *Rhodobacter sphaeroides*, the ATP binding site has not been definitely determined [60]. Therefore, it is assumed that the ε subunit from α-proteobacteria does not bind ATP. However, its CTD appears to be in the “down” conformation [44].

In mitochondria, when *pmf* drops during ischemia or during uncoupling, the hydrolytic activity of F_1_·F_o_ is inhibited by IF1 [39]. IF1 is a 10 kDa protein-forming dimer at acidic pH, that binds to the F_1_ part of F_1_·F_o_ [40]. The inhibition mechanism includes two steps: (i) *binding*, the NTD of IF1 enters through the open αE/βE catalytic interface; (ii) *blocking*, after the first catalytic turnover of F_1_·F_o_, rotation of γ by 120° leads to deeper incorporation of IF1, at the same time, the initially disordered NTD of IF1 undergoes a transition to an ordered structure in the form of an α-helix and mechanically inhibits further rotation of the mitochondrial F_1_·F_o_ [71,72]. The bacterial ε CTD binds to F_1_ at the same place where the IF1 subunit contacts the α and β subunits, blocking subunit γ rotation [40].

In most α-proteobacteria, exemplified by *P. denitrificans*, ATP hydrolysis appears to be controlled by another 11 kDa protein called the ζ subunit [17]. The presence of the ζ subunit in both *Pd*F_1_ and *Pd*F_1_∙F_o_ preparations indicates that it is an integral subunit of the enzyme [18]. Although the amino acid sequence of the ζ subunit is very different from that of the mitochondrial IF1 or bacterial ε subunit, its NTD has retained some similarity with the inhibitory domain of mitochondrial IF1 [18]. The conserved mobile NTD contains the inhibitory part of the protein, since the mutant lacking the first 14 residues of the NTD completely lost its inhibitory function, although it was still able to bind to *Pd*F_1_ [18]. ζ binds to *Pd*F_1_∙F_o_ via a bind/lock mechanism similar to that of IF1 in the mitochondrial F_1_·F_o_: The ζ subunit enters the open catalytic interface, allowing the γ subunit to make a 120° turn induced by ATP binding, then contacts the γ subunit and blocks its further rotation [44]. It is assumed that the structure of the ζ subunit is rigid enough to stop the further rotation of the γ subunit in the CCW direction. A low-affinity nucleotide binding site was found in the C-terminal part of the ζ subunit [18]. It is hypothesized that this site could work as an ATP sensor similar to the ε subunit sensor described in *E. coli* and *Bacillus* sp. PS3 [66] and regulate the binding affinity of ζ [18].

### 4.3. «Ratchet and Pawl» Mechanism of F_1_∙F_o_

**Figure 2:**
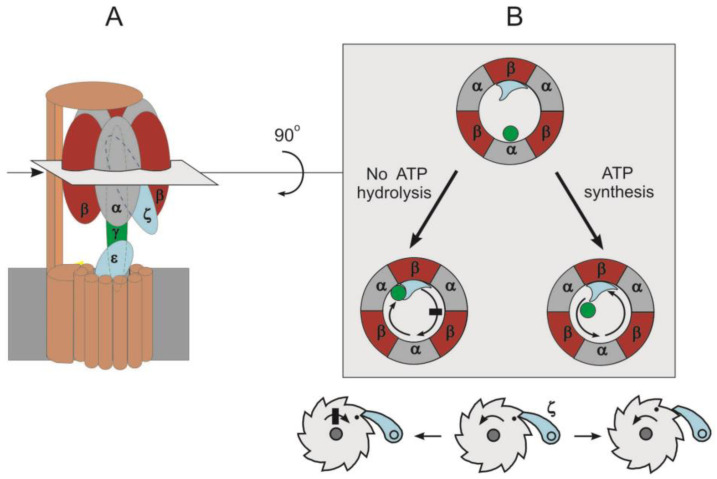
The inhibition of ATP hydrolysis in F_1_·F_o_ by inhibitor protein according to pawl-ratchet mechanism. (**A**): Schematic representation of F_1_·F_o_ (*P. denitrificans*). The subunit colors are the same as in Figure 1, but the peripheral stalk and *c*-ring are shown in brown and ζ subunit is colored in blue. The deep insertion of inhibitory protein (ζ subunit) into αβ interface is depicted. Cross-section through the F_1_ domain is shown. (**B**): View of F_1_ from the top; for clarity, the δ and *bb*_1_ subunits are not shown. Β and ζ subunits form a small indentation acting as pawl teeth. The pawl-ratchet mechanism enables the γ subunit’s free rotation in the ATP synthesis direction but stops rotation in ATP hydrolysis direction.

To avoid the wasting of cellular ATP, inhibitor proteins, ε, ζ, and IF1 work in a unidirectional manner, preventing only F_1_∙F_o_ hydrolase activity and not affecting the rate of ATP synthesis. To explain the unidirectional action of inhibitor proteins, it was suggested that they act like a ratchet and pawl mechanism (Figure 2), which was originally described for the bacterial ε subunit [73]. According to the modern view, the inhibitor protein acts like a pawl formed by γ/ε subunits, preventing CCW rotation of the rotor and, thereby, inhibiting ATP hydrolysis (mechanical ratchet). A mechanical obstacle, in the form of an inhibitor protein deeply inserted into 3α3β, can be overcome by *pmf* or by the substrate binding energy [72]. Alternatively, during ATP synthesis, F_1_∙F_o_ CW rotation is possible due to partial or complete release of the inhibitor protein (conformational pawl-ratchet) [72]. Experimentally, the ratchet mechanism is confirmed for the ε subunit. CryoEM maps show that the contact of the ε and β subunits in the βTP conformation blocks the rotation of the rotor in the direction of ATP hydrolysis, while it is still free to rotate in the direction of ATP synthesis. These data may explain the ability of the ε subunit to selectively inhibit ATP hydrolysis [16].

The ratchet mechanism is supposed to be universal for all known F_1_∙F_o_. A sequence of evolutionary events was proposed, suggesting that the change in the type of inhibitor protein that provides unidirectional inhibition of ATP hydrolysis did not essentially change the mechanism of inhibition. In *P. denitrificans*, the ε subunit lost its inhibitory function due to the deletion of the C-terminus and the loss of the ATP binding pocket [18,72]. ζ has evolved to replace ε as the main inhibitor of ATPase activity in free-living α-proteobacteria. However, the inhibitory function of ζ in some symbiotic α-proteobacteria was also partially lost, and also completely lost in some entirely parasitic α-proteobacteria, such as bacteria of the order *Rickettsiales*. *P. denitrificans* is described as the α-proteobacterium closest to mitochondria due to the similarity of their respiratory chains, which include four respiratory complexes [74]. Therefore, it is believed that in mitochondria resulting from endosymbiosis, IF1 arose independently through convergent evolution [75].

The ratchet mechanism of F_1_∙F_o_ inhibition by inhibitor proteins providing unidirectional inhibition of ATP hydrolysis is widely discussed in the literature [16,62,75,76,77]. However, some data are not consistent with this model [60].

Eubacteria with extremely low ATPase activity, in which F_1_∙F_o_ are only capable of ATP synthesis, are well known in the literature. Latent ATP hydrolysis activity has been shown, for example, for *Bacillus* species [78], *C. thermarum* [67], *M. smegmatis* [79], *Mycobacterium bovis* [80,81], and *P. denitrificans* [82]. However, inhibition by the ε subunit has been confirmed only in *Bacillus* species [60,78]. In *P. denitrificans* [44], *F. nucleatum* [3], and *M. smegmatis* [69], the ε subunit is in the “down” position, and inhibition of ATP hydrolysis by the ε subunit-mediated ratchet mechanism cannot be realized [60].

Moreover, *K_D_* of the ε·ATP complex differs significantly in different organisms and either exceeds (*E. coli*, 22 mM) [66], or is significantly below, the average physiological total ATP concentration in living cells (*Bacillus* sp. PS3, 0.29 μM) [83], which is in the millimolar range [84,85]. Thus, it is unlikely that ATP binding is involved in the regulation of ε subunit-mediated ATP hydrolysis under physiological conditions.

The role of the ε subunit in the regulation of bacterial ATP synthases is being actively studied using genetic modifications. On the one hand, it was shown that the ATPase activity and ATP-dependent proton-translocating activity of the *E. coli* F_1_∙F_o_ (*Ec*F_1_∙F_o_) lacking the ε subunit were reduced. These cells showed a reduced growth rate and lower viability in a low-salt medium [86]. It was also reported that the deletion of five residues in the εCTD reduced the ATP synthesis in *Ec*F_1_∙F_o_, leading to a decrease in the growth rate under aerobic conditions by about three times [87]. On the other hand, when the entire εCTD was genetically removed, *E. coli* did not show noticeable growth defects under a wide range of conditions in vitro [86]. Thus, the physiological significance of the ε subunit in the F_1_∙F_o_ regulation remains unclear.

Although ε has been shown to preferentially inhibit the ATPase activity without significantly affecting the ATP synthase activity of F_1_∙F_o_ [16,72,73], there is evidence that ε actually inhibits ATP synthesis [88]. Some researchers do not consider the ε subunit as a unidirectional inhibitor protein, but as a subunit that modulates the rotation rate of the rotor [64]. Thus, the mechanism of ε action has not been fully established.

Since its discovery, IF1 has been considered a unidirectional inhibitor of ATP hydrolysis [39]. However, there have been indications that IF1 also inhibits ATP synthesis. Thus, it was shown that IF1 can slow down both the pre-stationary phase and the steady-state ATP synthesis in mitochondria [89]. Studies of the IF1 function in various cell lines produced conflicting results, which did not help to understand the real role of this protein in the whole organism. In some experiments, IF1 knockout increased mitochondrial ATPase activity [61]. Other IF1 knockout mutants in yeast, mice or *Caenorhabditis elegans* showed no difference in growth, reproduction or bioenergetics compared to wild types [75].

The *P. denitrificans* mutants lacking the ζ subunit gene demonstrated a specific growth defect associated with an increase in *Pd*F_o_∙F_1_ ATP hydrolytic activity [62] in one case. However, in another work, the ζ subunit knockdown showed only a slight increase in ATP hydrolysis by *Pd*F_o_∙F_1_ [45].

Thus, a promising hypothesis of mechanical inhibition of ATPase by inhibitor proteins by the ratchet mechanism [72] does not fully explain the data set on the unidirectional regulation of F_1_∙F_o_ hydrolytic activity. Therefore, there are other factors to be taken into account.

It is well known that ATP hydrolysis in IF1-free mitochondrial F_1_∙F_o_ can be inhibited by ADP(Mg^2+^), but the inhibited enzyme will be fully active towards the ATP synthesis [90]. Furthermore, at least two factors are responsible for the inhibition of ATP hydrolysis in *P. denitrificans*, ζ subunit [18] and ADP(Mg^2+^) [46,54]. Their role in the inhibition of the hydrolytic activity of *Pd*F_1_ was confirmed in single-molecule experiments. It remains unclear which of these factors has a dominant effect on *Pd*F_o_∙F_1_ latent ATP hydrolysis [33]. In these experiments, significant differences were observed in the average lifetime of enzyme-inhibitor complexes, as well as in their ability for reactivation. While the inhibitory effect of ADP(Mg^2+^) has an average duration of about 30 s and is removed spontaneously, the period of inhibition mediated by the ζ subunit is extended by more than 500 s and is not spontaneously removed. The authors concluded that ADP(Mg^2+^) only modulates, while the ζ subunit completely blocks, the rotation of the *Pd*F_1_∙F_o_ in the hydrolytic direction [33]. On the other hand, in the *Pd*F_1_∙F_o_ double mutants, lacking both the ε-CTD and the ζ subunit, no significant activation of ATP hydrolysis was observed. Instead, even in double mutant strains, hydrolysis can only be activated by oxyanions, LDAO, or *pmf*, which are considered to remove ADP(Mg^2+^)-inhibition [91], which indicates the main role of ADP(Mg^2+^)-inhibition in the control of hydrolytic activity of the *P. denitrificans* F_o_∙F_1_.

Thus, the understanding of F_1_∙F_o_ unidirectional catalysis may require more complex schemes involving several regulatory mechanisms.

In this regard, both the mutual action of inhibitory proteins and ADP(Mg^2+^) [59,91,92] and their independent action on the F_1_∙F_o_ regulation are considered [50], but no consensus has been reached so far. Using compounds with a pronounced activating effect on the latent F_1_∙F_o_ hydrolytic activity, it was concluded that εCTD- and ADP(Mg^2+^)-induced inhibition are mutually exclusive for the membrane-bound *Ec*F_1_·F_o_ [50]. On the other hand, based on the rearrangement in the enzyme structure observed upon binding of ADP(Mg^2+^), it was suggested that the ε subunit can prevent the transition of F_1_∙F_o_ to the ADP(Mg^2+^)-inhibited state [25].

In any case, the trigger is needed to start the inhibitory mechanism. The ADP/ATP ratio or *pmf* were suggested to act as a trigger. However, ATP-dependent regulation of the ε subunit was shown not for all F_1_∙F_o_ [60]; it was not found for IF1 and only suggested for the ζ subunit [18]. In mitochondria, ADP(Mg^2+^) inhibits F_1_∙F_o_ at micromolar concentrations of the nucleotide [36]. In addition, F_1_∙F_o_ in different organisms show significant differences in the magnitude of ADP(Mg^2+^)-inhibition, which can change with variations in the concentration of P_i_ and nucleotides [37].

In addition to the ADP/ATP ratio, *pmf*, which can be considered a “substrate/product” of ATP synthesis/hydrolysis reactions, can also act as a factor regulating the inhibition of ATP hydrolysis [46]. For chloroplast [19], mitochondrial [52] and bacterial [53,54] enzymes, it was shown that membrane energization leads to the rapid activation of the ADP(Mg^2+^)-inhibited F_1_∙F_o_ hydrolytic activity. In single-molecule studies of *Bacillus* sp. PS3 F_1_, activation of the ADP(Mg^2+^)-inhibited enzyme was demonstrated upon mechanical rotation of the γ subunit [93]. In addition to the release of ADP(Mg^2+^) inhibition, it is believed that *pmf* induces conformational changes that remove inhibition caused by the εCTD action [55,94]. Recently, it was reported that the ε subunit changes its conformation not only in response to a change in the ATP concentration, but also in response to an increase in *pmf* [95].

It should be noted that most of the data on the regulation of F_1_∙F_o_ was obtained by studying the ATP-hydrolase activity of the water-soluble fragment, F_1_, or F_1_∙F_o_, in preparations that are unable to maintain the membrane potential. In order to study the role of *pmf* in the F_1_∙F_o_ regulation, it was necessary to introduce into research practice coupled membrane preparations that do not require artificial coupling and have a high respiratory control ratio. Sufficiently simple techniques [96] make it possible to obtain preparations of tightly coupled *P. denitrificans* membranes with high respiratory control, up to 4.5 when NADH is oxidized as a respiratory substrate [54], enabling the comparative study of ATP synthesis/hydrolysis and the role of membrane potential in the regulation of F_1_∙F_o_. The enzyme in *P. denitrificans* membranes has become a popular object of research at the present time [23,46,49,74,91,97].

## 5. *Paracoccus denitrificans* as a Unidirectional F_1_∙F_o_ Model

Although the *P. denitrificans* F_1_∙F_o_ has a common bacterial complex structure (3α:3β:γ:δ:ε:*a*:*b*:*b’*:12*c*) and one intrinsic inhibitor protein ζ [23], the enzyme also has unique properties. These are a very high rate of ATP synthase and a very low rate of F_1_- or F_1_∙F_o_-ATPase, with an ATP synthase/ATPase ratio of 20–120, which is more than 100 times higher than that of other bacteria, such as *E. coli* (ratio of 0.25) or mitochondrial F_1_∙F_o_ (ratio of 0.2) [62]. *Pd*F_1_∙F_o_ is considered a model of a unidirectional enzyme [46,82]. Establishing the mechanism of its regulation can help solve the problem of the unidirectional operation of F_1_∙F_o_ in general.

ATP hydrolase activity of *Pd*F_1_∙F_o_, in addition to the significant activation by sulfite [49], is also activated by *pmf* [54]. The studies of *Pd*F_1_∙F_o_ in tightly coupled inside-out sub-bacterial particles performed in Vinogradov’s group showed that *pmf* not only induces but also maintains the ATP hydrolase state of the enzyme [46,54]. It was also shown that neither an increase in ATP concentration, nor a decrease in P_i_ concentration—both causing a decrease in the ATP synthesis rate—affected the ATP/(ADP × P_i_) ratio maintained by coupled *P. denitrificans* particles at the end of ADP phosphorylation. A decrease in the respiratory chain activity also did not result in the ADP concentration remaining in the medium after the ATP synthesis was completed. The authors concluded that the mass action ratio, ATP/(ADP × P_i_), of the reaction catalyzed by F_1_∙F_o_, is not in equilibrium with *pmf* generated by respiration [98].

It is assumed that the chemical-mechanical relationship between ATP hydrolysis and F_1_ rotation is reversible, and during ATP synthesis, the order of structural changes in the enzyme accompanying ATP hydrolysis is reversed [1]. However, the question of whether ATP synthesis by the entire F_1_∙F_o_ complex is the exact mechanistic reversal of ATP hydrolysis reaction remains a subject of discussion [36,99]. If the synthesis and hydrolysis of ATP occur according to a single catalytic mechanism, then inhibitors and activators will have the same effect on the F_1_∙F_o_ forward and reverse reactions. However, a significant difference was found in the pH profiles of ATP synthesis and hydrolysis: a decrease in pH from 8.0 to 7.0 led to a reversible inhibition of ATP hydrolysis, while the activity of ATP synthesis did not change. Thus, the hydrolytic/synthetic activity of the enzyme is unidirectionally controlled by the pH [100].

Moreover, there are compounds that selectively affect the forward (synthesis) or the reverse reactions (hydrolysis) only. These are the so-called unidirectional enzyme inhibitors: aurovertin [101], azide [90], sulfite [102] and venturicidin [82,103]. The existence of such inhibitors is inconsistent with the concept of the simple reversibility of F_1_∙F_o_. However, the effect of unidirectional inhibitors was explained by the fact that *pmf* can affect the kinetics of the enzyme-inhibitor interaction [82] since the oxidative phosphorylation was measured in energized particles, while ATP hydrolysis was measured in uncoupled particles. Vinogradov’s group performed a detailed inhibitory analysis of *Pd*F_1_∙F_o_ ATP synthesis and hydrolysis, measuring ATP hydrolysis by the ATP-dependent reduction of NAD^+^ by succinate (reverse electron transfer), i.e., in energized membranes. Significant differences were found in the action of venturicidin, a specific inhibitor of bacterial F_1_∙F_o_, in energized membranes, depending on the direction of the reaction: (i) venturicidin was shown to inhibit ATP synthesis and ATP hydrolysis of *Pd*F_1_∙F_o_ but had an almost ten-fold difference in the affinity for the enzyme depending on the direction of catalysis; (ii) the synthesis of ATP was titrated almost linearly while for the hydrolysis of ATP, the titration produced a sigmoidal dependence [103].

### Hypothesis of Two Forms of F_1_∙F_o_: ATP Synthase and ATP Hydrolase

**Figure 3:**
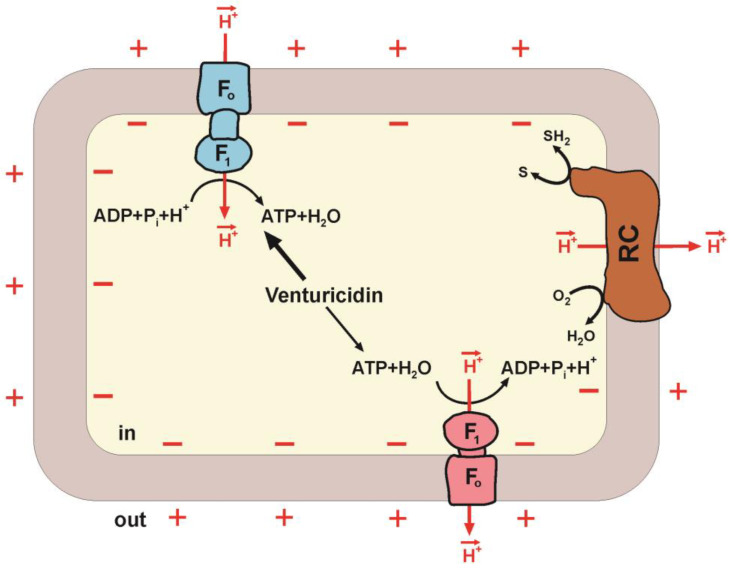
Synthesis and hydrolysis of ATP catalyzed by two forms of F_1_·F_o_. The main components of a bacterial cell coupling membrane are shown. The proton motive force (*pmf*) is generated by the respiratory chain (RC). F_1_∙F_o_ ATP synthase (blue) and ATP hydrolase (red) are composed of hydrophilic F_1_ that performs the catalytic function, and F_o_ provides proton translocation. F_o_ acts as a mechanical driving device rotating the γ subunit of F_1_. Venturicidin inhibition is also shown. Arrows in the centre indicate high affinity of ATP synthase (thick arrow) and low affinity of ATP hydrolase (thin arrow) for venturicidin.

The results obtained in Vinogradov’s group and other laboratories have led to the suggestion of models of the mechanism of oxidative phosphorylation based on the differences in the catalytic pathways of the forward and reverse reactions [36,99]. The development of this model led to the hypothesis, proposed by Vinogradov, according to which ATP synthesis and hydrolysis are catalyzed by two different *non-equilibrium* forms of F_1_∙F_o_ in the coupled energy-converting membranes—synthase and hydrolase [36,46]. The kinetic properties of the synthase form are best suited for efficiently controlled *pmf*-dependent ATP synthesis, and the second one is similarly adapted for ATP-dependent *pmf* generation [46] (Figure 3). In this model, two forms are understood as F_1_∙F_o_ complexes containing or not containing some subunits or, for example, differing in the set of specific annular phospholipids, and their lifetime is significantly greater than the time of catalytic turnover [46]. This hypothesis is in good agreement with experimental data [98,100,103].

The concept of two forms is supported by data from other groups. Thus, comparing the activating effects of sulfite and *pmf* on latent ATPase activity, it was hypothesized that both *pmf* and oxyanions activate different inhibited states present in the *Pd*F_1_∙F_o_ population [49]. The two-state hypothesis was used to explain the relationship between the three types of regulation (mediated by the ε and ζ subunits and ADP(Mg^2+^)-inhibition), suggesting that ε-CTD induces a change in the distribution of inhibited states, changing the proportion of the enzyme population capable of activation by *pmf* [91].

During the purification of *Pd*F_1_∙F_o_ for X-ray analysis, it was eluted from the Q HiTrap column with two separate peaks in comparable amounts, which were designated as F-ATPases I and II. It turned out that these forms differ in their ability to retain native lipids from the bacterial membrane [23]. The ATPase activity of the *P. denitrificans* mutants lacking ε and ζ was still latent and manifested only in the presence of sulfite or LDAO [91].

Subunit ε inhibits the ATPase activity incompletely, which is considered as not inhibition but modulation of rotor rotation. However, two forms of the enzyme, sensitive and insensitive to ε subunit upon ATP hydrolysis, could explain this observation [60]. Two forms of the enzyme are involved in the interpretation of data on two forms of regulation in *E. coli*—mediated by ε and ADP(Mg^2+^) [50]. It has recently been shown that venturicidin interacts better with active F_1_∙F_o_ form and worse with ADP(Mg^2+^)-inhibited enzyme [104]. The ratio between these forms is known to be controlled by *pmf* [53,54].

It was shown that the H^+^/ATP coupling ratio (the number of protons transferred across the membrane per one molecule of hydrolyzed ATP) in bacterial F_1_∙F_o_ (*Rb. capsulatus* and *E. coli*) depends on the ADP concentration [105]—a decrease in the ADP concentration at a constant ATP concentration was accompanied by a decrease in the number of H^+^ transferred per hydrolyzed ATP. To explain these data, the concept of two interconvertible states of ATP synthase, differing by their coupling ratios, was introduced. It has been suggested that the state with higher coupling ratios favors the binding of ADP, and the state with lower coupling ratios favors ATP binding [106].

These data can be explained by the model of two *non-equilibrium* forms of F_1_∙F_o_, unidirectionally catalyzing the synthesis and/or hydrolysis of ATP. These forms can be characterized by different types of regulation, which explains the variety of regulatory elements (ε, ζ, and ADP(Mg^2+^)) in one organism.

## 6. *Mycobacterium tuberculosis* F_1_∙F_o_ as a Promising Drug Target

Currently, F_1_∙F_o_ are actively considered as targets of antimicrobial agents [9,10,11,12,13,107], and various compounds well known as specific F_1_∙F_o_ inhibitors are considered candidates for this role [11,108]. It is particularly important that F_1_∙F_o_ is a new attractive target for medicines against the tuberculosis pathogen *M. tuberculosis* [14,109]. The absence of NAD-dependent lactate dehydrogenase in *M. tuberculosis* makes oxidative phosphorylation extremely important for its growth [110]. Therefore, for this bacterium, F_1_∙F_o_ is a vital enzyme necessary to provide unusually large amounts of ATP used for the synthesis of its cell wall [10,111].

### Bedaquiline Is Effective in Curing Highly Drug-Resistant Tuberculosis via Targeting M. tuberculosis F_o_∙F_1_

**Figure 4:**
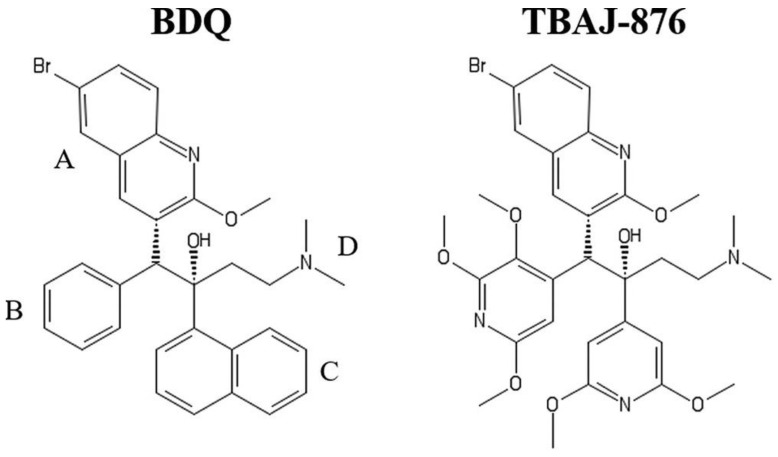
Structures of bedaquiline (BDQ) and TBAJ-876. In TBAJ-876, BDQ’s quinoline (A) and dimethylamino (D) groups are retained, whereas its phenyl (B) and naphthalene (C) groups are replaced with 2,3,5-trialkoxypyridin-4-yl and 3,5-dialkoxypyridin-4-yl groups, respectively. Reprinted from Sarathy et al. [122] under the terms of the Creative Commons Attribution 4.0 International Public License.

Tuberculosis is an infectious disease caused by the bacterium *M. tuberculosis*. It kills more people than any other infectious disease of bacterial origin. The emergence and spread of multidrug-resistant, extensively drug-resistant, and totally drug-resistant strains of *M. tuberculosis* is a great challenge in anti-tuberculosis treatment [112,113]. Thus, there is an urgent need to create potent antimycobacterial agents with a novel mechanism of action. The development of compounds that target energy metabolism enzymes in *M. tuberculosis*, such as the respiratory chain complexes and F_1_∙F_o_, is now considered a new promising strategy. Bedaquiline (TMC207, BDQ, Sirturo™) was the first drug approved by the U.S. Food and Drug Administration (FDA) and the European Medicines Agency (EMA), which belongs to the class of bioenergetics inhibitors [114,115,116,117]. It is a diarylquinoline derivative having a quinolinic central heterocyclic nucleus with alcohol and amine side chains (Figure 4, left structure) which are suggested to play a significant role in anti-tuberculosis activity [115,118]. Bedaquiline was reported to selectively target F_1_∙F_o_ of *M. tuberculosis* by interacting with the F_o_ domain [14,119,120]. This leads to the inhibition of ATP production and a substantial decrease in ATP levels [119,121]. The bactericidal effect of bedaquiline was observed with both replicating and dormant bacterial subpopulations [119,121].

The mycobacterial F_1_∙F_o_ is composed of nine different subunits with a stoichiometry of 3*α*:3β:*γ*:*δ*:*ε*:*a*:*b*:*b’*:9*c* [123,124]. The membrane-embedded subunit *a* and the rotating *c*-ring transfer protons from the intermembrane space to the cytoplasm through two half-channels in subunit *a* [6,124,125]. These half-channels are separated by an essential arginine residue in subunit *a*, which interacts with a key, proton-translocating glutamate residue of the *c*-ring, causing the protonation change [126,127].

Similar to the *P. denitrificans* enzyme, mycobacterial F_1_·F_o_ is active in ATP synthesis but hydrolyzes ATP at very low rates, and their latent ATP hydrolase activity is activated by the *pmf* [79]. Although the exact mechanism of mycobacterial F_1_·F_o_ regulation has not yet been established, inhibition of ATP hydrolase activity [124] is thought to result from the interaction of a unique C-terminal extension of the α subunit and 14 additional amino acid residues of the γ subunit [80,81,128]. It was also shown that the duplicated domain in the N-terminal region of the fused bδ subunits can interact with the N-terminal region of the α subunit [125], blocking the rotation of the rotor in the CW direction. In addition, it is believed that the ε subunit [129], as well as hydrolysis products, ADP and P_i_, that surprisingly have been found in the βE catalytic site, contribute to the ATP hydrolysis inhibition [69].

**Figure 5:**
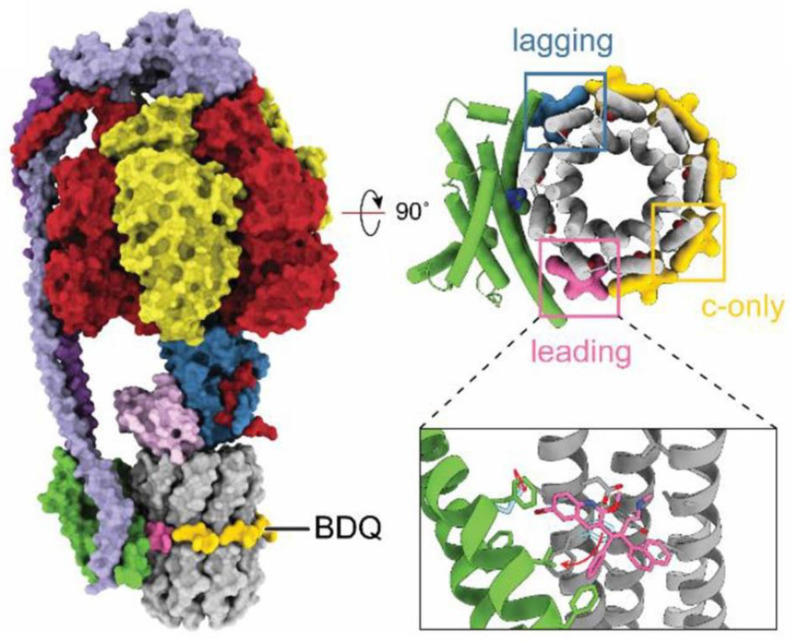
Structure of *M. smegmatis* F_1_·F_o_ ATP synthase bound to bedaquiline (PDB ID: 7JGC). Bedaquiline (BDQ) binds at five *c*-only sites (yellow), a leading site (pink), and a lagging site (blue) in the F_o_ region of the enzyme. Red arrows show the movement of residues upon bedaquiline binding. Adapted from Courbon and Rubinstein [6] under the terms of the Creative Commons Attribution 4.0 International Public License.

The cryoEM structure of the *M. smegmatis* F_1_·F_o_ shows seven bedaquiline binding sites in the F_o_ domain and large-scale conformational changes induced in the enzyme by the inhibitor (Figure 5) [6,124]. Five bedaquiline molecules bind with a lower affinity to the *c*-ring. In these subunit *c*-sites (denoted as ‘*c*-only sites’), the dimethylamino group of bedaquiline interacts with the carboxyl group of the proton-carrying glutamate-65 residue. Two more molecules of bedaquiline bind with a higher affinity to two respective subunit *a*/*c*-interfaces. The latter two sites are designated as the ‘leading site’ and the ‘lagging site’. The ‘leading site’ involves a subunit *c* that has just interacted with subunit *a* and picked up a proton from the periplasm. The ‘lagging site’ involves a subunit *c* poised to interact with subunit *a* to deposit a proton into the cytoplasm [124]. Thus, the wedge-like binding of bedaquiline to the two subunit *a*/*c*-interfaces blocks the rotation of the F_1_·F_o_ rotor [124,127]. Hards et al. [130] proposed a second inhibition mechanism upon investigating a blockage of ATP synthesis by bedaquiline in *E. coli* inside-out membrane vesicles. The mechanism is based on the ability of bedaquiline (a weak lipophilic base, pK_a_ = 8.9), upon its localization at F_1_·F_o_, to function as a H^+^/K^+^ ionophore, thus uncoupling the oxidative phosphorylation. This specific and potent uncoupling thus leads to the dissipation of *pmf* and equilibration of transmembrane pH and potassium gradients [130]. The findings reported by Sarathy et al. [122], however, are in disagreement with that work. They suggest that the uncoupler activity is not required for diarylquinolines to exert their antimycobacterial activity. Further studies are needed to clarify the discrepancy.

The successful therapeutic advance of bedaquiline was, however, overshadowed by the observation of acquired resistance of *M. tuberculosis* to the drug [14,131]. Furthermore, recently, it has become known that bedaquiline also potently inhibits the yeast and human mitochondrial F_1_·F_o_ [132], despite reports of the contrary [8,133,134]. The site of bedaquiline inhibition was found to partially overlap with that of oligomycin. Surprisingly, molecular dynamics simulations suggest that the binding mode of bedaquiline to this site is similar to that previously observed for a mycobacterial enzyme [132]. Although Luo et al. noted that “the discrepancies between this and other studies of the inhibition of BDQ of the human enzyme are due to differences in the assays and methods, and in some cases, also due to species and cell-specific effects” [132], the potential risks associated with taking the drug because of that discovery cannot be ignored. In view of the particular importance of the issue, further research is required in this direction. One more drawback of bedaquiline is its very high lipophilicity, which may contribute to its extremely long elimination half-life and tissue accumulation at high concentrations [118]. Another drawback of the drug is its inhibitory action (IC_50_ = 1.6 µM) on the cardiac potassium channel protein encoded by the human ether-a-go-go-related gene (hERG) [135]. Dysfunction of the hERG channel causes long QT syndrome and increases the risk of sudden death in patients with cardiac ischemia [118]. The development of next-generation analogs of bedaquiline, which would have the potential to address the above-mentioned issues, is clearly necessary. A newly developed 3,5-dialkoxypyridine analog of bedaquiline named TBAJ-876 (Figure 4, right structure) is currently in phase II trials. Compared to bedaquiline, TBAJ-876 displays improved pharmacological and toxicological properties [118] but retains the same mycobactericidal activity [122]. A new class of selective and potent inhibitors of the mycobacterial F_1_·F_o_ appeared to be tetrahydronaphthalene amides (THNAs). THNAs are effective in preventing the growth of *M. tuberculosis* in culture and show improved hERG liability, clearance, and half-life compared to bedaquiline [136].

In light of the above in this and the previous sections, when developing new drugs, it is necessary to take into account not only the F_1_∙F_o_ structure, but also the complexity of its regulatory mechanisms. For example, it has been found that although venturicidins do not exhibit antibacterial activity, they are able to enhance the action of aminoglycoside antibiotics against various bacterial pathogens [137]. However, a recent study of *Ec*F_1_∙F_o_ showed that the ATPase activity inhibited by low concentrations of venturicidin is restored after prolonged incubation with the inhibitor at high concentrations. [104]. A similar effect was observed in experiments with F_1_∙F_o_ from *M. smegmatis*, which was inhibited by about 80% by nanomolar bedaquiline, but most of the activity was restored by micromolar bedaquiline. [124].

At the end of this section, it should be noted that the use of a specific and potent inhibitor of the mycobacterial F_1_∙F_o_, in combination with inhibitors of other bioenergetics enzymes of *M. tuberculosis*, such as cytochrome *bd* and/or a *bcc*-*aa*_3_ supercomplex, may have a synergistic effect [113,138,139,140,141,142]. This would represent an innovative pharmaceutical strategy for the treatment of highly drug-resistant tuberculosis.

## 7. Concluding Remarks

Various bacterial F_1_∙F_o_ complexes are structurally very similar but show significant differences in the regions of the structure responsible for enzyme regulation [3]. The variety of regulatory elements (ε, ζ, and ADP(Mg^2+^)) may provide different regulatory pathways, but in our opinion, they may also belong to different forms of the enzyme [46,103]. Although X-ray diffraction analysis and cryoEM are two powerful research methods for studying the interaction of drug molecules with F_1_∙F_o_, such approaches cannot take into account the role of *pmf* in that interaction. We believe that the hypothesis of two F_1_∙F_o_ forms, and the use of F_1_∙F_o_ preparations which allow taking into account the role of *pmf*, may be useful for establishing the mechanisms of F_1_∙F_o_ regulation and, further, for biomedical research.

## Data Availability

Data sharing not applicable.
